# Uncovering sensory axonal dysfunction in asymptomatic type 2 diabetic neuropathy

**DOI:** 10.1371/journal.pone.0171223

**Published:** 2017-02-09

**Authors:** Jia-Ying Sung, Jowy Tani, Tsui-San Chang, Cindy Shin-Yi Lin

**Affiliations:** 1 Department of Neurology, Wan Fang Hospital, Taipei Medical University, Taipei, Taiwan; 2 Department of Neurology, School of Medicine, College of Medicine, Taipei Medical University, Taipei, Taiwan; 3 Ph.D. Program for Neural Regenerative Medicine, College of Medical Science and Technology, Taipei Medical University and National Health Research Institutes; 4 Neural Regenerative Medicine, College of Medical Science and Technology, Taipei Medical University and National Health Research Institutes; 5 Translational Neuroscience, Department of Physiology, School of Medicine Science, Faculty of Medicine, University of New South Wales, Sydney, Australia; University of Toronto, CANADA

## Abstract

This study investigated sensory and motor nerve excitability properties to elucidate the development of diabetic neuropathy. A total of 109 type 2 diabetes patients were recruited, and 106 were analyzed. According to neuropathy severity, patients were categorized into G0, G1, and G2+3 groups using the total neuropathy score-reduced (TNSr). Patients in the G0 group were asymptomatic and had a TNSr score of 0. Sensory and motor nerve excitability data from diabetic patients were compared with data from 33 healthy controls. Clinical assessment, nerve conduction studies, and sensory and motor nerve excitability testing data were analyzed to determine axonal dysfunction in diabetic neuropathy. In the G0 group, sensory excitability testing revealed increased stimulus for the 50% sensory nerve action potential (P<0.05), shortened strength-duration time constant (P<0.01), increased superexcitability (P<0.01), decreased subexcitability (P<0.05), decreased accommodation to depolarizing current (P<0.01), and a trend of decreased accommodation to hyperpolarizing current in threshold electrotonus. All the changes progressed into G1 (TNSr 1–8) and G2+3 (TNSr 9–24) groups. In contrast, motor excitability only had significantly increased stimulus for the 50% compound motor nerve action potential (P<0.01) in the G0 group. This study revealed that the development of axonal dysfunction in sensory axons occurred prior to and in a different fashion from motor axons. Additionally, sensory nerve excitability tests can detect axonal dysfunction even in asymptomatic patients. These insights further our understanding of diabetic neuropathy and enable the early detection of sensory axonal abnormalities, which may provide a basis for neuroprotective therapeutic approaches.

## Introduction

Type 2 diabetes mellitus (DM) is an alarming health concern worldwide [1]. Among its complications, diabetic neuropathy is a major cause of morbidity in DM, and may affect up to 50% of long-standing diabetic patients. Sensory symptoms are much more prominent than motor in typical diabetic neuropathy [2]. It is known that the majority of patients have distal symmetrical peripheral neuropathy [3], and neuropathic pain has a detrimental impact on quality of life [4, 5].

Despite recent evidence suggesting that intensive therapy might reduce the risk of developing diabetic neuropathy, once it has developed, even strict glycemic control cannot reverse neuropathic symptoms and pathological changes [3]. This fact underlines the importance of early detection and treatment of diabetic neuropathy.

Many aspects of the pathogenesis of diabetic neuropathy remain to be explored, but recently, a number of well conducted studies have broadened our understanding on the subject [6–9]. Although the exact molecular basis underlying diabetic neuropathy is complex, metabolic alterations such as glucose toxicity, alteration of insulin receptors, glucose uptake and utilization may affect neurons early in the disease process. These metabolic alterations would lead to ATP depletion, mitochondrial dysfunction, and changes in ion conductance [9]. These defects would then set the stage for further structural and functional defects, eventually compromising axonal integrity and function. Recent evidence also suggests that sensory symptoms in diabetic patients may be related to dysregulated ion channel expression in sensory axons [10–12].

Nerve excitability testing is a useful tool to provide further understanding regarding the pathogenesis of diabetic neuropathy. Previously, we demonstrated that the test can provide valuable electrophysiological data that added to our understanding of how diabetes causes dysfunction in motor nerves. It was also able to detect motor axonal dysfunction in diabetic patients even before the onset of diabetic neuropathy [13]. Nevertheless, as sensory symptoms are typically more prominent than motor symptoms [2], an assessment of sensory nerve excitability could provide even more important insights into the pathogenesis of diabetic neuropathy from a nerve excitability viewpoint. It also has the potential to provide greater sensitivity in the detection of early axonal dysfunction.

## Materials and Methods

Clinical assessments, conventional nerve conduction studies (NCS), and nerve excitability testing were performed in patients with type 2 DM. All the patients met the American Diabetic Association criteria for diabetes diagnosis [4]. Patients with carpal tunnel syndrome, cervical radiculopathy, myopathy, hyperkalemia/hypokalemia, or with other potential causes for sensory polyneuropathy such as vitamin B12 deficiency, alcohol abuse, uremia, or autoimmune diseases were excluded based on clinical assessments and NCS results. All patients enrolled in the study were recruited from the Wan Fang Hospital, Taipei Medical University, Taipei, Taiwan. Control nerve excitability data were obtained from 33 healthy control (HC) subjects that were age- and sex-matched with the patients’ cohort. The study was approved by the Joint Institution Review Board of Taipei Medical University. All the subjects gave their signed informed consent for inclusion in the study.

### Clinical assessments and NCS

**Fig 1:**
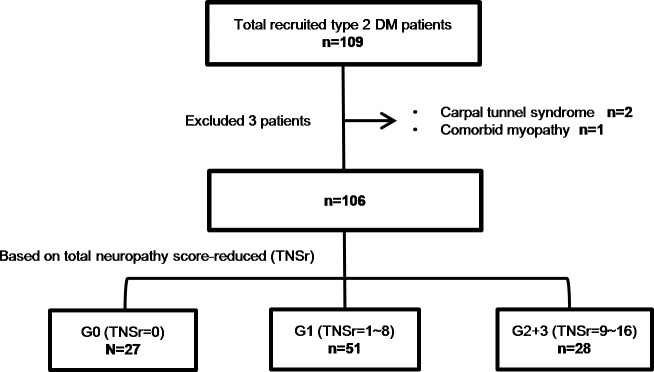
This flowchart depicts the recruitment and the subjects involved in the final data analysis. Patients were divided into group G0, G1, and G2+3 based on total neuropathy score-reduced (TNSr).

Standard neurological examinations were performed for all patients. To quantify the neuropathic symptoms, the TNSr was obtained in each patient [14]. The reduced version of the total neuropathy score used is focused on the symptomatic presentation. The scoring criteria include the extent and severity of the following subscore items: sensory symptoms, motor symptoms, autonomic symptoms, deficit in pin sensibility, deficit in vibration sensibility, and deficit in muscle strength with a score of 0 corresponding to no symptoms and a score 4 to severe symptoms in each criterion. Patients were then categorized into three grades based on the TNSr, corresponding to neuropathy severity: grade 0 (G0) with no symptoms (TNSr 0), grade 1 (G1) with mild symptoms (TNSr 1–8), and grade 2+3 (G2+3) with moderate/severe symptoms (TNSr 9–24). A detailed patient classification scheme can be seen in Fig 1.

Conventional NCSs were performed for all subjects. To determine the diagnostic utility of sensory nerve excitability test in early diabetes, we compared nerve excitability test parameters of HCs with patients who had yet to develop clinically relevant NCS changes for diabetic neuropathy, as defined in criterion 3 of the American Academy of Neurology diagnostic criteria [15]. Routine blood tests, glycosylated hemoglobin (HbA1c) level, and serum creatinine level were also obtained.

### Nerve excitability testing

Nerve excitability studies were performed by stimulating the nerve median at the wrist according to previously described protocols, with skin temperature over the wrist maintained at least 32.0 degrees Celsius [16, 17]. Paired recordings of motor and sensory nerve excitability indices were obtained for each subject. Sensory nerve action potentials (SNAPs) were recorded from the index finger, while compound muscle action potentials (CMAPs) were recorded from the abductor pollicis brevis muscle. Stimulation and recording were controlled by software (QTRAC version 28/10/2011; Institute of Neurology, London, U.K.) and the stimulus current was administered using an isolated linear bipolar constant-current stimulator (DS5; Digitimer, Welwyn Garden City, U.K.). Surface electrodes were used during recording. The changes in current required to produce a target potential corresponding to 50% of the maximal CMAP or SNAP were tracked. Latency was defined as the time delay (ms) between stimulus onset and peak CMAP or SNAP response. The stimulus threshold was defined as the current (mA) that was required to produce amplitudes of CMAP or SNAP responses of half maximal amplitude.

The nerve excitability protocol incorporated the following recordings: 1) a stimulus response (SR) curve; 2) strength-duration (SD) relationship which also determined rheobase and strength-duration time constant (SDTC); 3) threshold electrotonus (TE) utilizing subthreshold 100-ms polarizing currents in both depolarizing (TEd; +40%) and hyperpolarizing (TEh; -40%) directions to change the potential difference across the internodal membrane; and 4) recovery cycle (RC) using a paired pulse paradigm with a supramaximal conditioning stimulus followed by a test stimulus at interstimulus intervals from 2 to 200 ms. Superexcitability was measured as the maximal threshold reduction, and subexcitability as the maximal threshold increase after an interstimulus interval of 10 ms.

### Statistical analysis

Nerve excitability data of diabetic patient groups and HCs were compared with one-way ANOVA with post hoc analysis. Correlation studies were performed with Pearson R. Data analysis was performed using the Statistical Package for the Social Sciences (SPSS) for Windows version 21 (SPSS Inc., Chicago, U.S.A.).

## Results

We obtained adequate sensory nerve excitability testing results from 109 patients. Of these, 2 were excluded for having carpal tunnel syndrome, and 1 was excluded for having comorbid myopathy (Fig 1). The demographics and clinical profiles for the 106 patients are shown in Table 1. They were further subdivided by TNSr score: 27 patients were categorized in the G0 group, 51 were categorized in the G1 group, and 28 patients in the G2+3 group. Although the mean body weight of diabetic patients was higher than healthy controls, the increment was not statistically significant. Compared to the G0 group, G1 and G2+3 groups had longer diabetes duration since diagnosis.

**Table 1:** Patient demographics and clinical and electrophysiological profiles.

Variable	Healthy controls	Type 2 diabetes		
		G0	G1	G2+3
TNSr (score)	-	0(0.00)	4.06(2.28)	11.46(2.05)
Sex: male/female (number)	14/19	9/18	26/25	18/10
Age (yr.)	62.11(7.51)	61.19(8.15)	62.37(12.90)	63.00(10.85)
HbA1c (mg/dl)	5.90(0.51)	7.12(0.76)***	7.77(1.42)***	7.82(1.76)***
Creatinine (mg/dl)	0.75(0.16)	0.78(0.24)	0.87(0.32)*	1.26(1.73)
Body weight (kg)	61.07 (10.21)	65.89 (10.80)	67.34 (12.68)	68.81 (12.09)
Diabetes duration (yr. since diagnosis)	N/A	3.27 (3.39)	5.79 (4.31)	6.48 (4.48)
Diabetes medications	N/A	Insulin (8%), Biguanides (58%), SU (16%), Acarbose (13%), Meglitinides (3%), DPP-4i (34%), Thiazolidinediones (0%)	Insulin (22%), Biguanides (76%), SU (27%) Acarbose (5%), Meglitinides (2%), DPP-4i (27%), Thiazolidinediones (0%)	Insulin (19%), Biguanides (67%), SU (48%), Acarbose (10%), Meglitinides (0%), DPP-4i (29%), Thiazolidinediones (5%)

The reported values of laboratory data represent the mean (standard deviation).

TNSr = Total neuropathy score-reduced [14], SU = sulfonylureas, and DPP-4i = dipeptidyl peptidase 4 inhibitors.

*t-test P<0.05 vs. healthy controls and

***t-test P<0.001 vs. healthy controls.

A summary of conventional NCS results is shown in Table 2. A trend of decreasing amplitude and nerve conduction velocity could be observed in sural and median nerves. The mean sural NCV data (41.03 m/s) in the G2+3 group was abnormal when compared to the normal range of this laboratory (41.5–58.3 m/s).

**Table 2:** Summary of conventional nerve conduction study results.

Variable	Healthy controls	Type 2 diabetes		
		G0	G1	G2+3
Sural NCV (m/s)	60.27(7.97)	54.00(7.79)	49.25(12.29)	41.03(18.46)*
Sural amplitude (μV)	16.33(6.17)	15.46(5.61)	14.19(9.73)	8.96(8.16)
Median sensory NCV (m/s)	57.88(8.86)	55.15(5.64)	51.43(6.61)	49.59(8.06)
Median sensory amplitude (μV)	35.39(14.26)	31.94(16.50)	28.94(10.58)	22.00(10.22)
Median motor NCV (m/s)	55.64(4.31)	52.89(11.68)	51.71(5.30)	50.28(5.39)
Median motor amplitude (mV)	7.47(2.63)	7.36(2.60)	7.44(2.08)	7.25(1.98)

The reported values of laboratory data represent the mean (standard deviation).

NCV = nerve conduction velocities.

*The mean data are out of normal range for this NCS laboratory. The normal ranges in this NCS laboratory were: sural NCV 41.5–58.3 m/s, sural amplitude 5–37.16 μV, median sensory NCV 48.7–65.5 m/s, median sensory amplitude 10.0–72.6 μV, median motor NCV 49.2–64.8 m/s, and median motor amplitude 3.0–15.4 mV.

**Fig 2:**
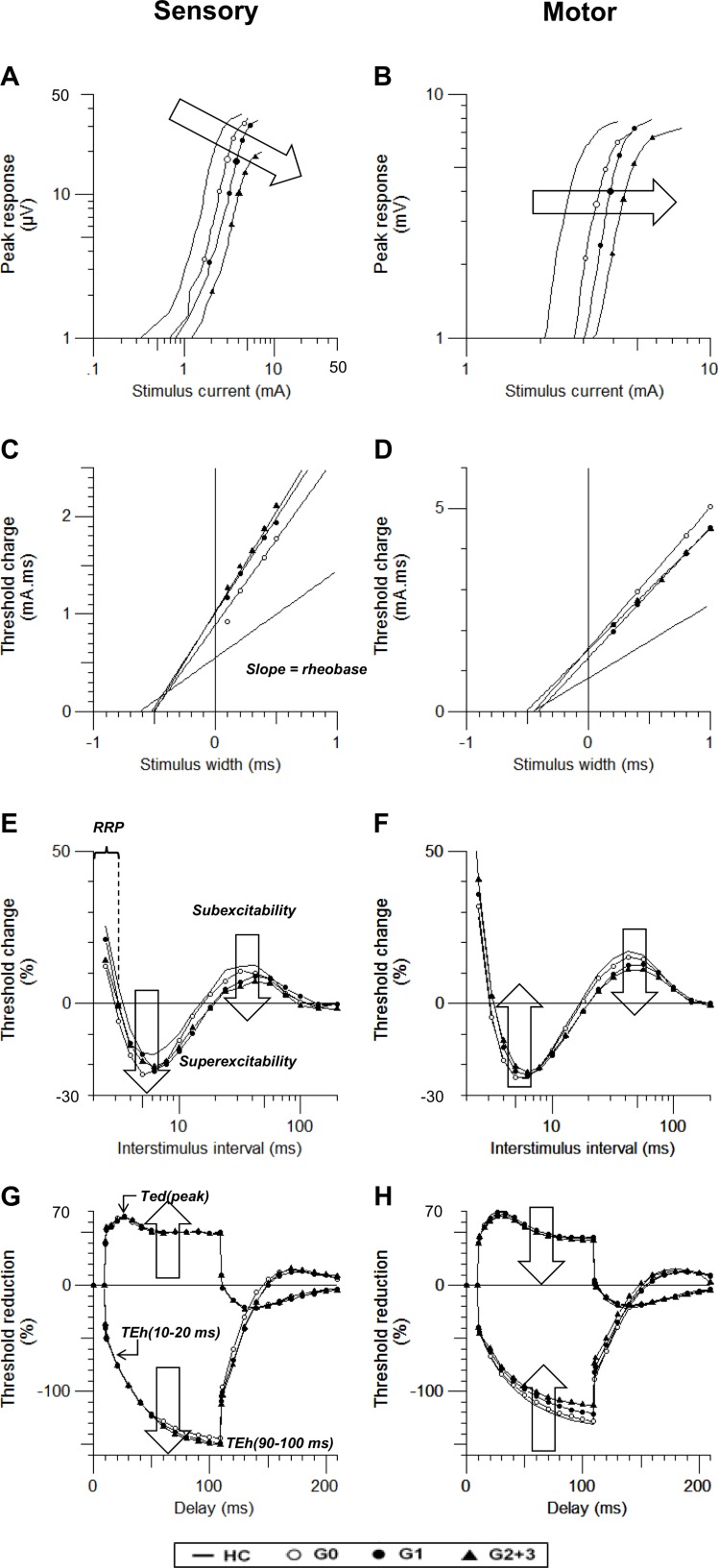
**(A and B)** Comparison of the stimulus response curve, **(C and D)** strength-duration time constant, **(E and F)** recovery cycle, and **(G and H)** threshold electrotonus in diabetic patients (G0: empty circle, G1: filled circle, and G2: triangle) and healthy controls (line). Sensory profiles are shown in the left column, while motor profiles are shown in the right.

Sensory and motor axonal excitability indices in patients are listed in Table 3 and Fig 2. In the G0 cohort, sensory excitability indices in patients differed from HCs in the stimulus for 50% SNAP, SDTC, superexcitability, subexcitability, and TEd(peak). However, motor excitability indices showed significant changes only in stimulus for 50% CMAP.

**Table 3:** Comparison of sensory and motor nerve excitability parameters between groups.

Axonal properties	HC (n = 33)	Type 2 diabetic patients (n = 106)			P value (F)✝
		G0 (n = 27)	G1 (n = 51)	G2+3 (n = 28)	
**Sensory stimulus-response curve**					
Stimulus for 50% SNAP (mA)	2.24±0.18	3.66±0.58**	4.06±0.22***	4.27±0.27***	<0.001 (8.44)
Peak response (μV)	39.36±2.47	37.96±3.19	38.79±2.94	24.00±2.52***	<0.001 (7.52)
Latency (ms)	3.33±0.06	3.51±0.06	3.87±0.11***	3.87±0.07***	<0.001 (8.11)
Sensory SDTC (ms)	0.62±0.02	0.54±0.02**	0.55±0.02**	0.55±0.03*	0.011 (3.87)
**Sensory recovery cycle**					
RRP	3.44±0.12	3.28±0.12	3.43±0.12	3.36±0.12	NS (0.28)
Superexcitability (%)	-16.61±1.06	-22.65±1.50**	-22.70±1.28**	-21.53±1.41*	0.004 (4.65)
Subexcitability (%)	12.39±0.61	10.46±0.57*	9.64±0.53**	8.22±0.78***	<0.001 (7.38)
**Sensory threshold electrotonus**					
TEd(peak) (%)	59.36±0.51	62.59±0.90*	62.44±0.65**	63.10±1.36**	0.016 (3.54)
TEh(90–100 ms) (%)	-145.44±4.35	-143.68±5.43	-148.54±3.17	-150.08±4.46	NS (0.41)
**Motor stimulus-response curve**					
Stimulus for 50% CMAP (mA)	2.76±0.14	5.33±0.81***	4.73±0.28***	5.70±0.30**	<0.001 (7.18)
Peak response (mV)	8.31±0.46	7.94±0.97	10.17 ±2.33*	9.80±0.54	NS (2.16)
Latency (ms)	6.62±0.17	6.78±0.16	6.98±0.09	6.93±0.17	NS (1.45)
Motor SDTC (ms)	0.46±0.01	0.47±0.02	0.44±0.01	0.53±0.02**	0.001 (5.65)
**Motor recovery cycle**					
RRP	3.12±0.09	3.15±0.12	3.33±0.09	3.47±0.12*	NS (2.27)
Superexcitability (%)	-23.98±0.98	-24.07±1.76	-22.56±0.97	-21.80±1.43	NS (0.72)
Subexcitability (%)	16.44±0.94	14.47±0.81	12.40±0.57***	11.15±0.68***	<0.001 (9.23)
**Motor threshold electrotonus**					
TEd(peak) (%)	68.33±0.69	66.85±1.16	67.25±0.84	63.99±1.11**	0.022 (3.31)
TEh(90–100 ms) (%)	-129.87±3.82	-127.22±5.00	-120.50±3.04	-112.91±3.07**	0.014 (3.66)

The reported values represent the mean ± standard error.

HC = healthy control; SNAP = sensory nerve action potential; CMAP = compound muscle action potential; SDTC = strength-duration time constant; RRP = relative refractory period; and NS = not statistically significant.

✝P values and F from one-way ANOVA between groups.

*Post hoc analysis P<0.05 vs. healthy controls

**Post hoc analysis P<0.01 vs. healthy controls, and

***Post hoc analysis P<0.001 vs. healthy controls.

Comparison of sensory and motor SR curve is shown in Fig 2A and 2B. Both sensory (stimulus for 50% SNAP, 3.66±0.58 mA; P < 0.01) and motor (stimulus for 50% CMAP, 5.33±0.81 mA; P < 0.001) SR curve shows a right shifting of the curve in the G0 group compared to that of HC, and the trend persisted into the G1 (sensory: 4.06±0.22 mA; P < 0.001, motor: 4.73±0.28 mA; P < 0.001), and G2+3 groups (sensory: 4.27±0.27 mA; P < 0.001, motor: 5.70±0.30 mA; P < 0.01). This indicates that both sensory and motor axons have increased thresholds. In addition, in SNAP peak response decreased as the disease progressed in the G2+3 group (24.00±2.52 μV; P < 0.001), suggesting axonal loss in the G2+3 group. One-way ANOVA confirmed increased stimulus for 50% SNAP (P < 0.001, F = 8.44) and CMAP (P < 0.001, F = 7.18), decreased sensory peak response (P <0.001, F = 7.52), and increased sensory latency (P <0.001, F = 8.11) across groups.

The sensory and motor SD relationship can be seen in the threshold charge vs stimulus width plot in Fig 2C and 2D. Sensory SDTC in the diabetic group were shorter than that of HCs (G0 group: 0.54±0.02 ms; P < 0.01, G1 group: 0.55±0.02 ms; P < 0.01, G2+3 group: 0.55±0.03 ms; P < 0.05). The motor SDTC of patients showed prolongation late in G2+3 group (0.53±0.02 ms; P < 0.01). One-way ANOVA showed results consistent with shortening of sensory SDTC (P = 0.011, F = 3.87) and prolongation of motor SDTC (P < 0.001, F = 9.23) across diabetic patient groups.

The results of RC are shown in Fig 2E and 2F. In sensory axons, increased superexcitability (-22.65±1.59%; P < 0.01) and decreased subexcitability (10.46±0.57%; P < 0.05) from the G0 group were noted. Changes in superexcitability remained as the disease progressed further (G1 group: -22.70±1.28%; P < 0.01; G2 group: -21.53±1.41%; P < 0.05). Subexcitability also remained decreased into the advanced stage of the disease (G1 group: 9.64±0.53%; P < 0.01, G2+3 group: 8.22±0.78%; P < 0.001). One-way ANOVA also supported increased subexcitability (P = 0.004, F = 4.65) and decreased superexcitability (P < 0.001, F = 7.38) in diabetic groups. There was a trend of shortening of the mean RRP in later diabetic neuropathy (G2+3 group: 3.36±0.12 ms).

In agreement with the previous study by Sung et al., 2012, the motor RC curve showed no significant difference in superexcitability. The motor subexcitability was significantly decreased in the G1 group (12.40±0.57%; P < 0.001) and worsened as the disease progressed (G2+3 group: 11.15±0.68%; P < 0.001), a change confirmed by one-way ANOVA between groups (P < 0.01, F = 9.23). The motor mean RRP also showed a trend of prolongation in the late stage (G2+3 group: 3.47±0.12).

The plotting of sensory and motor TE is shown in Fig 2G and 2H. The sensory TE showed progressive axonal dysfunction from G0 to the G2+3 group. Under depolarizing conditioning current, sensory TE showed increasing TEd(peak) (G0 group: 62.59±0.90%; P < 0.05, G1 group: 62.44±0.65%; P < 0.01, G2 group: 63.10±1.36%; P < 0.01). Although insignificant, there was a trend for decreased accommodation toward hyperpolarizing current in the later stage of disease, which could be seen as “fanning-out” of TEh(90–100 ms) (G0 group: -143.68±5.43%; G1 group: -148.54±3.17%; G2+3 group: -150.08±4.46%). The “fanning-out” of TEd(peak) across groups was supported by one-way ANOVA (P = 0.016, F = 3.54).

TE in motor axons showed a gradual “fanning-in” in later stages of diabetes both toward depolarizing and hyperpolarizing current. “Fanning-in” toward depolarizing current was seen in TEd(peak) showing more significant decrement in the G2+3 group (63.99±1.11%; P < 0.01). In addition, “fanning-in” toward hyperpolarizing current could be seen as TEh (90–100 ms) showing changes in the G2+3 group (-112.91±3.07%, P < 0.01). One-way ANOVA results were consistent with “fanning-in” in TEd(peak) (P = 0.022, P = 3.31) and TEh(90–100 ms) (P = 0.014, F = 3.66) across groups.

### Nerve excitability in patients not meeting diabetic neuropathy criteria

**Fig 3:**
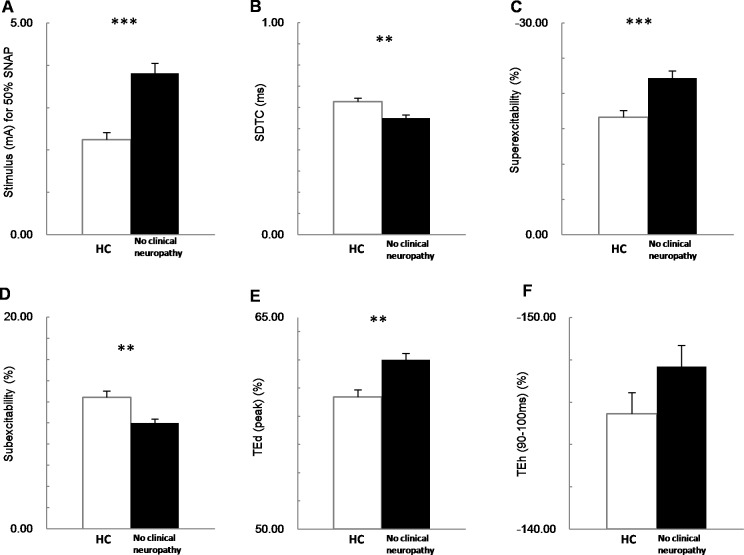
The difference between healthy control (HC, white bar) and type 2 diabetic patients who have no clinical neuropathy (no clinical neuropathy, black bar) in sensory nerve excitability parameters. **(A)** No clinical neuropathy group shows greater threshold stimulus for 50% SNAP, **(B)** shorter strength-duration time constant (SDTC), **(C)** increased superexcitability, **(D)** decreased subexcitability, **(E)** greater peak of TEd, and **(F)** the TEh in a 90–100 ms time window. *P<0.05; ** P<0.01; *** P<0.001

To assess the early changes in sensory nerve excitability indices of patients prior to developing conventional NCS changes, we compared the sensory excitability indices of normal controls (n = 33) with patients who had not yet developed clinically relevant NCS changes for diabetic neuropathy (n = 78) using an unpaired t-test. There are several significant differences between HCs and the patients in their sensory nerve excitability indices (Fig 3), including: elevated stimulus for 50% SNAP, (3.41±1.05 vs. 2.25 ±0.18 mA; P < 0.001), prolonged latency (3.65±0.04 vs. 3.33±0.06 μV; P < 0.001), shortened SDTC (0.55±0.01 vs. 0.62±0.02 ms; P < 0.01), increased superexcitability (-22.2±0.90 vs. -16.61±1.06%; P < 0.001), increased subexcitability (9.99±0.38 vs. 12.39±0.61%; P < 0.01), and increased TEd(peak) (61.99±0.47 vs. 59.36±0.51%; P < 0.01). These results reveal that diabetic sensory axonal excitability was altered prior to developing clinically relevant changes in NCS.

### Correlation studies between clinical parameters and nerve excitability

**Fig 4:**
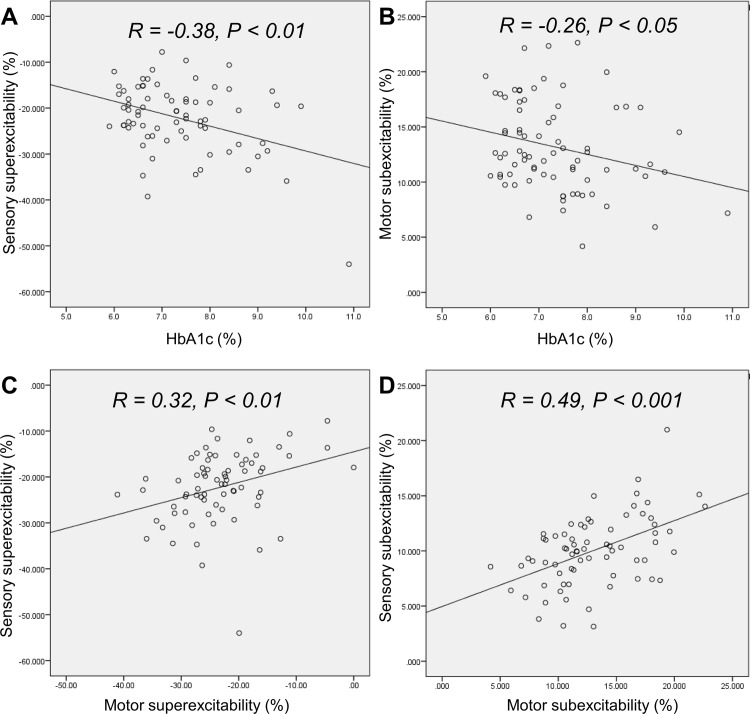
Correlation analysis in patients without clinically relevant neuropathy (n = 78). **(A)** Correlation between sensory superexcitability and HbA1c level. **(B)** Correlation between motor subexcitability and HbA1c level. **(C)** Correlation between sensory and motor superexcitability parameters. **(D)** Correlation between sensory and motor subexcitability parameters.

The correlations between HbA1c and nerve excitability parameters were investigated in patients who had not yet developed clinical neuropathy. The results of the analysis show correlations between HbA1c level and motor subexcitability (P<0.05, R = -0.26) as previously reported [13]; sensory superexcitability (P< 0.001, R = -0.38) and latency (P<0.05, R = 0.23) both correlate with HbA1c level (Fig 4A and 4B).

Furthermore, in all diabetic patients, longer duration since diabetic diagnosis was correlated with increased latency in sensory (P<0.05, R = 0.24) and motor axons (P<0.05, R = 0.24), and increased motor TEh(90–100 ms) (P<0.05, R = 0.21)

To further dissect the relationship between sensory and motor axons in the same DM patient, the correlation between paired sensory and motor excitability parameters was also evaluated: sensory superexcitability was statistically correlated to motor superexcitability (P <0.01, R = 0.32) (Fig 4C) and motor TEd(peak) (P<0.05, R = -0.27). Where sensory subexcitability also correlated to motor subexcitability (P < 0.001, R = 0.49) (Fig 4D), the results confirmed concurrent changes of the motor and sensory excitability parameters in the same patients.

## Discussion

This study is the first to explore the progression of diabetic neuropathy in sensory axons in asymptomatic patients with severe diabetic neuropathy, using an array of examinations including standard neurological examinations to quantify the neuropathic symptoms, TNSr, and nerve excitability testing. Substantial insights into the underlying pathophysiological mechanisms were obtained utilizing these tests. The findings suggest that sensory axons developed nerve dysfunction prior to and in a different fashion than that of motor axons. These striking results enable us to detect abnormalities in sensory axons at asymptomatic stages. In the following section, the mechanisms involved and underlying voltage-gated ion channel functions that may contribute to the differences between sensory and motor axons and the phenotype of diabetic neuropathies will be discussed.

### Nerve excitability dysfunction reflects the pathogenesis of diabetic neuropathy

**Fig 5:**
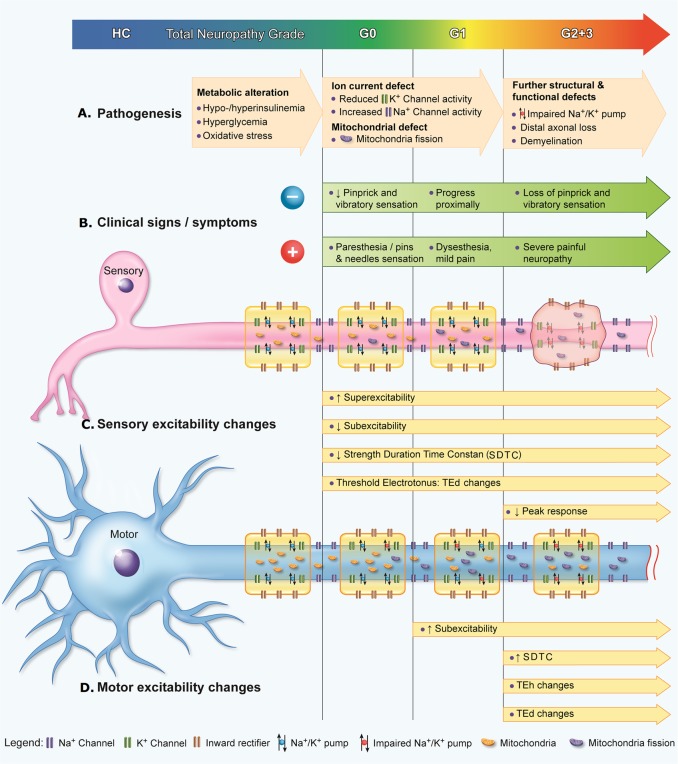
Progression of diabetic neuropathy from pathophysiologic, symptomatologic, and nerve excitability viewpoints. **(A)** Pathogenesis of diabetic neuropathy typically progresses from metabolic alteration, to ion current defect, and then the development of further structural and functional defects. **(B)** Both positive and negative clinical signs/symptoms would also progress in extent and severity as diabetic neuropathy worsens. **(C)** Sensory excitability changes, reflecting sensory axonal dysfunction, could be detected even in asymptomatic patients. Superexcitability, subexcitability, SDTC, and TEd parameter changes progress over the course of diabetic neuropathy, and eventually the peak response decreases, reflecting axonal loss. **(D)** Motor excitability changes in superexcitability, SDTC, TEh, and TEd parameters could be detected in later stages of diabetic neuropathy compared to sensory axons.

Abnormal excitability parameters developed over the course of the disease, reflecting gradual progression from metabolic alteration, to impairment of ion conductances, to further structural defects including dysfunction of the Na^+^/K^+^ pump, culminating in distal axonal degeneration [9]. (Fig 5A)

This study indicates that sensory axons develop axonal dysfunction earlier than motor axons: more sensory nerve excitability parameters were changed in the early stage in the G0 cohort, reflecting Na^+^/K^+^ pump or nodal Na^+^ channel impairment in the beginning of diabetic neuropathy. In contrast to motor axons, only increased stimulation threshold and decreased subexcitability were noted in G0. Sensory axons are liable to damage caused by hyperglycemia and mitochondrial dysfunction [9]. In G2+3 group, sensory axons eventually developed distal axonal loss, as evidenced by reduced peak amplitude; however, in the same group, motor axons did not develop significant axonal loss. Our findings support the concept that sensory axons are more sensitive to pathological changes in diabetes, compatible with the fact that sensory symptoms usually arise earlier than motor [3].

### Differences in the development of sensory and motor axonal dysfunction

Hofmeijer and colleagues found that sensory axons were more vulnerable to ischemia and hyperglycemic hypoxia than motor axons, presumably due to the greater sensory nerve dependency on the Na^+^/K^+^ pump [18]. Pump activity is also physiologically higher in sensory axons than in motor axons [19]. It has also been established that various biophysical differences in sensory and motor axons might lead to their different excitability properties in healthy as well as in diseased states [20, 21]. By studying the same patients’ motor and sensory axons, the present results indicate that excitability changes in sensory axons occur earlier and progress in a different fashion to motor axons.

In sensory axons of DM patients, right shifting of the SR-curve, shortening of SDTC, down-shifting of RC curve, together with a trend of RRP shortening and TE “fanning-out,” suggested that the sensory axon is possibly in a state of hyperpolarization [22]. These changes in excitability indices are also similar to changes observed in sensory axon during the post-ischemic period [23].

Sensory axons might be hyperpolarized at the early stage of DM due to the reduction of Na^+^ conductances in sensory axons, which could produce hyperpolarization. Reduction of nodal Na^+^ permeability has been observed in later stages of diabetic neuropathy in animal models [24–26]. This decrease in Na^+^ permeability might be due to progressive redistribution of nodal Na^+^ channels across the paranodal barrier into the paranodal and internodal domains [24]. Na^+^ channel expression might also be affected in DM by post-translational modification [27].

This study has shown that SDTC was progressively shortened, reflecting a decrement of persistent Na^+^ conductance in diabetes. Transient Na^+^ channels were probably also affected, as a trend of shortening in RRP was observed as the disease progressed, which could be responsible for the parathesia that patients suffer.

The motor nerve excitability dysfunction seen in this study are in agreement to those previously reported [7, 13, 28, 29]. The right-shifting of the SR curve, flattening of RC curve, and “fanning-in” of TE parameters are compatible with a reduction of Na^+^/K^+^ pump activity [7, 29]. The reduction of the pump activity itself might be related to either hyperglycemia, increased levels of sorbitol with consequent myo-inositol depletion, and/or reduction in protein kinase C activation [13]. Nerve sonographic study has revealed diffuse median nerve swelling from the wrist to forearm segment; the nerve swelling and the resultant ischemia might have affected the median nerve excitability indices in this study [30, 31].

### Correlation between nerve excitability and clinical parameters

Previous studies found evidence that glycemic control is associated with changes in axonal properties [28, 32–34]. Furthermore, membrane property changes were found to be correlated with neuropathy-specific quality-of-life measures and severity [35, 36]. This study found that higher HbA1c levels were correlated with increasing superexcitability and decreasing subexcitability in sensory axons of patients without clinically relevant neuropathy. This suggest that in patients with higher HbA1c, the inhibitory function of paranodal potassium channels might be weaker and that nerve excitability techniques can be a useful tool for future study on the effect of blood sugar control on nerve function, in particular, parameters such as motor subexcitability and sensory superexcitability. Positive correlation between diabetic duration and latency in motor and sensory axons indicated that axonal dysfunction tended to worsen during the later stages of diabetes.

### Concurrent changes in paired motor and sensory excitability

Correlation between numerous sensory and motor excitability parameters in the paired sensory and motor study confirmed concurrent pathological sensori-motor axonal changes in the same patients. While correlation between sensory superexcitability and motor superexcitability suggested that nodal and internodal potassium channels are affected in the same patients, the correlation between sensory subexcitability and motor subexcitability suggested slow potassium channels in the paranodal region might also be similarly affected [37]. Previous studies had suggested that potassium channels are affected by ischemia in both motor and sensory axons [37, 38].

### Possible implications for early neuroprotective therapeutic approaches

This study revealed that sensory nerve excitability indices showed axonal dysfunction earlier than motor. Sensory nerve excitability testing could be a potential screening tool for the early detection of peripheral nerve involvement. Among all parameters, sensory superexcitability appeared to be the most sensitive in the detection of early sensory axonal dysfunction. Clearly, more studies are needed to further establish the diagnostic utility of sensory excitability indices in diabetic neuropathy. Earlier detection of sensory axonal dysfunction would prompt earlier neuroprotection for diabetic neuropathy. Taken together, insights from this study provide a basis for new therapeutic approaches aimed at delaying or reversing diabetic neuropathy with the potential to further change clinical practice such that early neuroprotection can be initiated.
